# The clinical utility of Nanopore 16S rRNA gene sequencing for direct bacterial identification in normally sterile body fluids

**DOI:** 10.3389/fmicb.2023.1324494

**Published:** 2024-01-09

**Authors:** Hiu-Yin Lao, Lily Lok-Yee Wong, Yan Hui, Timothy Ting-Leung Ng, Chloe Toi-Mei Chan, Hazel Wing-Hei Lo, Miranda Chong-Yee Yau, Eddie Chi-Man Leung, River Chun-Wai Wong, Alex Yat-Man Ho, Kam-Tong Yip, Jimmy Yiu-Wing Lam, Viola Chi-Ying Chow, Kristine Shik Luk, Tak-Lun Que, Franklin Wang Ngai Chow, Gilman Kit-Hang Siu

**Affiliations:** ^1^Department of Health Technology and Informatics, The Hong Kong Polytechnic University, Kowloon, Hong Kong SAR, China; ^2^Department of Clinical Pathology, Pamela Youde Nethersole Eastern Hospital, Chai Wan, Hong Kong SAR, China; ^3^Department of Microbiology, Prince of Wales Hospital, Shatin, Hong Kong SAR, China; ^4^Department of Pathology, Princess Margaret Hospital, Kowloon, Hong Kong SAR, China; ^5^Department of Clinical Pathology, Tuen Mun Hospital, Tuen Mun, Hong Kong SAR, China

**Keywords:** Nanopore sequencing, 16S rRNA gene sequencing, direct bacterial identification, body fluids, rapid diagnosis

## Abstract

The prolonged incubation period of traditional culture methods leads to a delay in diagnosing invasive infections. Nanopore 16S rRNA gene sequencing (Nanopore 16S) offers a potential rapid diagnostic approach for directly identifying bacteria in infected body fluids. To evaluate the clinical utility of Nanopore 16S, we conducted a study involving the collection and sequencing of 128 monomicrobial samples, 65 polymicrobial samples, and 20 culture-negative body fluids. To minimize classification bias, taxonomic classification was performed using 3 analysis pipelines: Epi2me, Emu, and NanoCLUST. The result was compared to the culture references. The limit of detection of Nanopore 16S was also determined using simulated bacteremic blood samples. Among the three classifiers, Emu demonstrated the highest concordance with the culture results. It correctly identified the taxon of 125 (97.7%) of the 128 monomicrobial samples, compared to 109 (85.2%) for Epi2me and 102 (79.7%) for NanoCLUST. For the 230 cultured species in the 65 polymicrobial samples, Emu correctly identified 188 (81.7%) cultured species, compared to 174 (75.7%) for Epi2me and 125 (54.3%) for NanoCLUST. Through ROC analysis on the monomicrobial samples, we determined a threshold of relative abundance at 0.058 for distinguishing potential pathogens from background in Nanopore 16S. Applying this threshold resulted in the identification of 107 (83.6%), 117 (91.4%), and 114 (91.2%) correctly detected samples for Epi2me, Emu, and NanoCLUST, respectively, in the monomicrobial samples. Nanopore 16S coupled with Epi2me could provide preliminary results within 6 h. However, the ROC analysis of polymicrobial samples exhibited a random-like performance, making it difficult to establish a threshold. The overall limit of detection for Nanopore 16S was found to be about 90 CFU/ml.

## 1 Introduction

Invasive bacterial infections refer to the isolation of bacterial pathogens from normally sterile body fluids (Lee et al., 2011). These infections often present as clinical emergencies, particularly in cases of meningitis, pneumonia, and bloodstream infection. The prompt identification of the causative agents is crucial for providing effective treatment and reducing the mortality rates. Currently, culture remains the standard method for pathogen identification in clinical laboratories. However, it takes at least 24 h to obtain isolated colonies on solid media for microbial identification. In the case of blood cultures, the incubation time is even longer due to the low presence of circulating microbes in blood (Opota et al., 2015). Typically it takes 24 to 48 h to obtain a positive result in the blood culture system, and for some fastidious species, it may take up to 5 days (Kirn and Weinstein, 2013; Ransom et al., 2021). After that, an additional 24 to 48 h are required to obtain isolated colonies on solid medium.

Although Matrix-assisted laser desorption/ionization-time of flight mass spectrometry (MALDI-TOF MS) has reduced the time required for bacterial identification (Tsuchida et al., 2020), fresh isolated colonies are still necessary for accurate identification. The presence of human cells or multiple microorganisms in direct specimens can interfere with the protein spectra, leading to identification failures in MALDI-TOF MS. Moreover, the low quantity of microbes in direct specimens makes it more challenging to achieve correct identification using MALDI-TOF MS (Ferreira et al., 2011). A study by Chien et al. (2016) showed that only 72.1% (263/365) of monomicrobial blood cultures were correctly identified to the species level by MALDI-TOF MS when using direct blood cultures. For polymicrobial blood cultures, only 5% (2/40) were correctly identified both microorganisms at the species level. Additionally, only 35.1% (142/405) of samples had a confidence score ≧2.000 (Chien et al., 2016). Another study that used MALDI-TOF MS to directly identify microbes from CSF showed that only 38.6% (17/44) of samples were correctly identified (Bishop et al., 2018). Therefore, culture remains essential for accurate and reliable MALDI-TOF MS identification.

16S rRNA gene sequencing is commonly used as the reference method to determine the definitive identity of the unidentifiable species in MALDI-TOF MS (Church et al., 2020). The long-read Nanopore sequencing is advantageous for sequencing the entire 16S rRNA gene, which allows better taxonomic resolution at the species level. A previous study conducted by our group demonstrated that Nanopore sequencing achieved a diagnostic accuracy of 96.36%, which was similar to that of Sanger sequencing, for bacterial identification using 16S rRNA gene sequencing in clinical isolates (Lao et al., 2022). Due to its high sensitivity, real-time analysis platform, and the ability to identify mixed species, Nanopore 16S rRNA gene sequencing (Nanopore 16S) of direct specimens could be a potential alternative for rapid bacterial identification in clinical laboratories. Although prior studies have documented the utilization of Nanopore 16S for pathogen detection in clinical samples, many of the investigations often had small sample sizes or were confined to specific sample type. For instances, Nanopore 16S based pathogen detection in 8 CSF samples (Moon et al., 2019), 36 synovial fluids (Han et al., 2022), 31 culture-negative clinical samples (Bouchiat et al., 2022), 6 corneal specimens (Omi et al., 2022), 23 intraocular fluids (Low et al., 2022), and 32 FFPE neuropathology specimens (Albers et al., 2023) were reported. Additionally, there is no consensus on the cut off of relative abundance for differentiating the pathogens from contaminants in Nanopore 16S.

This study was aimed to evaluate the performance of Nanopore 16S in identifying pathogens in various types of normally sterile body fluids and establish a threshold of relative abundance (T_*RA*_) for discriminating potential pathogens from sequencing noises. A total of 213 normally sterile body fluids were collected and sequenced, including 128 cases of monomicrobial infections, 65 cases of polymicrobial infections and 20 culture-negative samples. To minimize classification bias, taxonomic classification was performed using 3 analysis pipelines: Epi2me, Emu, and NanoCLUST. The sequencing result was compared to the culture references and T_*RA*_ was determined based on the ROC analysis. The limit of detection of Nanopore 16S was also determined using simulated bacteremic blood samples, which were spiked with *Staphylococcus aureus* and *Klebsiella pneumoniae* respectively, with final concentrations of 10, 50, 100, and 150 CFU/ml.

## 2 Materials and methods

### 2.1 Sample collection and preparation

A total of 213 leftover body fluids, along with their corresponding culture results, were collected from the clinical microbiology laboratories of four public hospitals in Hong Kong: Pamela Youde Nethersole Eastern Hospital, Prince of Wales Hospital, Princess Margaret Hospital, and Tuen Mun Hospital. Detailed information regarding the specimen types and culture results are shown in Supplementary Table 1. Upon receival, DNA extraction was performed using QIAamp BiOstic Bacteremia DNA Kit.

### 2.2 16S rRNA sequencing

Library preparation was performed using 16S Barcoding Kit 1 – 24 (SQK-16S024) from Oxford Nanopore Technologies (ONT) according to the manufacturer’s protocol with some modifications. The suggested input is 10 ng of genomic DNA in the protocol. Since it is impossible to quantify only bacterial DNA in clinical samples with high human DNA background, the maximum input volume of 15 μl was added instead of 10 ng. The PCR cycle number was also increased from 25 to 35 cycles in order to increase the sensitivity of the assay. A total of 24 barcoded libraries were pooled in equal concentration and sequenced for up to 24 h using the flow cell FLO-MIN106 R9.4.1 with the sequencer GridION on the MinKNOW platform, with super-accuracy basecalling model. To reduce index misassignment, “mid-read barcode filtering” and “barcode both ends” were adopted, and the minimum barcoding score was set to be 85.

### 2.3 Sequencing data analysis

The sequencing reads were uploaded to Epi2me, the cloud-based analysis platform developed by ONT, for real-time analysis. The FASTQ 16S workflow (v2021.09.09) in Epi2me was adopted with a minimum QSCORE of 10, which is the default minimum QSCORE of super-accuracy basecalling model. The reported average read accuracy of R9.4.1 flow cell ranges from 92.3 to 96.52% in recent studies (Ni et al., 2023; Zhao et al., 2023), which is lower than the traditional 97% similarity cutoff to differentiate different species. Considering the relatively lower read accuracy of Nanopore sequencing, a minimum coverage and a minimum identity of 90% were adopted and only reads between 1,000 to 2,000 bps were included for the analysis. The sequencing reads were further analyzed with two additional pipelines: Emu (Curry et al., 2022) and NanoCLUST (Rodriguez-Perez et al., 2021), with default parameters.

### 2.4 Data and statistical analysis

The concordance between culture and the Nanopore 16S, coupled with the three analysis pipelines in culture-positive samples were calculated by (number of cultured species detected by Nanopore 16S)/(total number of cultured species). For the culture-negative samples, if clinically important species were obtained in Nanopore 16S, the results would be correlated with the clinical manifestations and medical history of the patients. All the statistical analysis was performed using GraphPad Prism (v9.5.0), a *p*-value less than 0.05 was regarded as statistically significant. The statistical difference between two populations of sequencing reads and classified species were calculated using Mann–Whitney U test.

### 2.5 Determination of threshold of relative abundance for detecting potential pathogens

Relative abundance of a species was calculated by (number of reads of a species in a sample)/(total number of classified reads of a sample). To determine the threshold of relative abundance (T_*RA*_) for detecting potential pathogens in body fluids using Nanopore 16S, a receiver operating characteristic (ROC) curve was illustrated for each analysis pipeline based on the relative abundance of true positives (detected by both culture and Nanopore 16S) and false positives (detected only by Nanopore 16S) using GraphPad Prism (v9.5.0). ROC analyses of monomicrobial and polymicrobial samples were performed separately. The optimal point with the maximum Youden’s index in the ROC curve was considered the T_*RA*_ for detecting pathogens in each analysis pipeline, and the average value of the three pipelines was considered the T_*RA*_ in Nanopore 16S.

### 2.6 LOD

To determine the LOD of Nanopore 16S, simulated bacteremic blood samples were prepared by spiking two reference strains (*Staphylococcus aureus* BAA-3114 and *Klebsiella pneumoniae* BAA-3079) in EDTA-blood provided by a healthy individual with a final concentration of 150, 100, 50, and 10 CFU/ml. The simulated samples were extracted and sequenced as authentic samples and analyzed using Epi2me. The experiment was repeated twice to eliminate random errors. The average relative abundances of each species in Nanopore 16S were plotted against the bacterial concentrations. A trend line was used to determine the minimal bacterial concentration that meets the T_*RA*_.

## 3 Results

### 3.1 Common bacterial pathogens in body fluids

Among the 128 monomicrobial body fluids, the most frequently identified pathogen in culture was coagulase-negative *Staphylococci* (21/128), followed by *Staphylococcus aureus* (20/128), *Escherichia coli* (12/128), and *Enterococcus faecalis* (12/128). Among the 65 polymicrobial body fluids, the most common isolated pathogen in culture was *Escherichia coli* (42/65), followed by *Klebsiella pneumoniae* (16/65) and *Bacteroides fragilis* (14/65). For Nanopore 16S, the twelve most abundant species and their respective relative abundances of each sample classified by the three analysis pipelines were summarized in Supplementary Table 2.

### 3.2 Statistics of Nanopore 16S sequencing reads

After 24-h sequencing, the monomicrobial samples yielded an average of 76,551 reads per sample (s.d. ± 91,867) with a median of 32,699 reads. The polymicrobial samples had comparatively more reads per sample than the monomicrobial samples (*p* < 0.0001), with an average of 160,172 reads (s.d. ± 168,470) and a median of 127,662 reads per sample. The culture-negative samples had significantly lower number of reads compared to those culture-positive samples (*P* < 0.0001), with an average of 23,442 reads (s.d. ± 54,352) and a median of 1,373 reads per sample.

### 3.3 Concordance between traditional culture and Nanopore 16S

**FIGURE 1:**
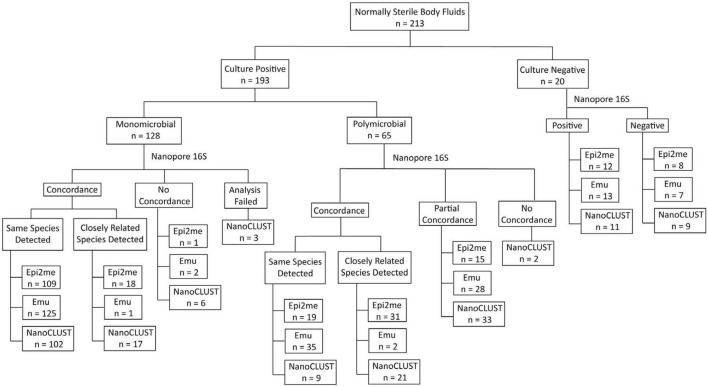
The overview of concordance between culture and Nanopore 16S coupled with the three analysis pipelines.

An overview of the concordance between culture and Nanopore 16S results was illustrated in Figure 1, while Supplementary Tables 3, 4 record the concordance of each monomicrobial and polymicrobial sample, respectively. In both monomicrobial and polymicrobial samples, Nanopore 16S coupled with Emu demonstrated the highest concordance. Among the 128 monomicrobial samples, Emu correctly identified the taxon of 125 (97.7%) samples, compared to 109 (85.2%) samples and 102 (79.7%) samples identified by Epi2me and NanoCLUST, respectively. Among the 65 polymicrobial samples, Emu correctly identified all the cultured species in 35 (53.8%) samples, compared to 19 (29.2%) samples and 9 (13.8%) samples in Epi2me and NanoCLUST, respectively.

Historically, a threshold of 97% sequence similarity in the 16S rRNA gene was used to differentiate two species (Janda and Abbott, 2007; Beye et al., 2018; Johnson et al., 2019). However, in some closely related species, the sequence similarity can be 98% or higher (Kawamura et al., 1995; Beye et al., 2018; Devanga Ragupathi et al., 2018). Supplementary Table 5 provides a list of closely related species (sequence similarity ≥ 98%). If those closely related species were considered concordant classifications, the number of correctly detected samples increased for Epi2me. In this case, the number of correctly detected samples for Epi2me, Emu, and NanoCLUST in monomicrobial samples increased to 127 (99.2%), 126 (98.4%), and 119 (93.0%), respectively. For the 65 polymicrobial samples, Epi2me successfully detected all the cultured species in 50 samples (76.9%), compared to 37 samples (56.9%) and 30 samples (46.2%) in Emu and NanoCLUST, respectively. Partially concordant samples were defined as those that failed to detect all but at least one of the cultured species by Nanopore 16S. In Epi2me, Emu, and NanoCLUST, a total of 15 samples (23.1%), 28 samples (43.1%), and 33 samples (50.8%) of polymicrobial samples were partially concordant with the culture results, respectively.

**FIGURE 2:**
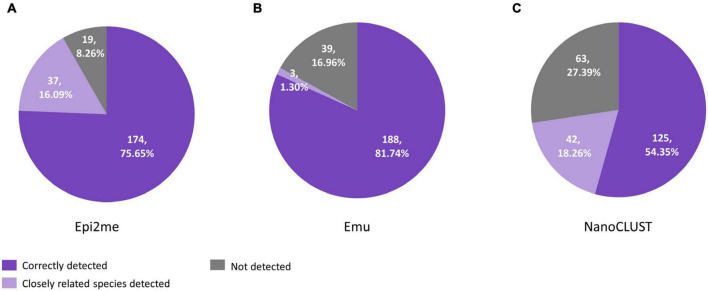
The concordance between culture and Nanopore 16S coupled with panel **(A)** Epi2me, **(B)** Emu and **(C)** NanoCLUST, in 230 cultured species among the 65 polymicrobial body fluids.

A total of 230 species were cultured from 65 polymicrobial body fluids. When considering all the cultured species in polymicrobial body fluids together, Nanopore 16S coupled with Emu showed the highest concordance (81.7%) if closely related species were not considered, compared to 75.7% for Epi2me and 54.3% for NanoCLUST (Figure 2). If closely related species were considered concordantly classified, Epi2me showed the highest concordance (91.7%), followed by Emu (83.0%), and NanoCLUST (72.6%).

**FIGURE 3:**
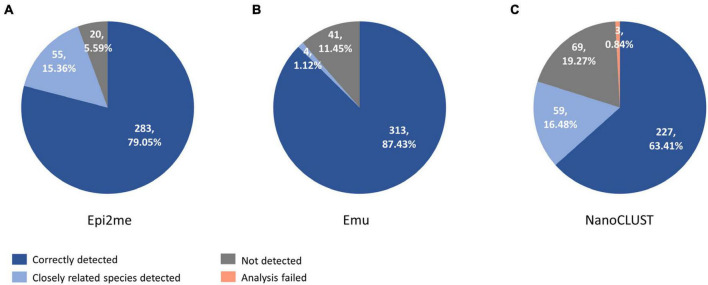
The concordance between culture and Nanopore 16S coupled with panel **(A)** Epi2me, **(B)** Emu and **(C)** NanoCLUST, in 358 cultured species among the 193 culture-positive body fluids.

The overall concordance of Nanopore 16S with culture in the 193 culture-positive body fluids, totaling 358 cultured species, was summarized in Figure 3. The overall concordance of Epi2me, Emu, and NanoCLUST was 79.1, 87.4, and 63.4%, respectively. If closely related species were considered concordantly classified, the overall concordance of Epi2me, Emu, and NanoCLUST would be 94.4, 88.6, and 79.9%, respectively.

In both monomicrobial and polymicrobial samples, there were cases where Nanopore 16S failed to detect the expected species based on the culture reference or their closely related species. In monomicrobial samples, the number of discordant samples was 1 (0.8%) for Epi2me, 2 (1.6%) for Emu, and 6 (4.7%) for NanoCLUST. Notably, three monomicrobial samples could not be analyzed by NanoCLUST, possibly due to insufficient reads (<120 reads) for cluster generation. For polymicrobial samples, two completely discordant samples were found solely in NanoCLUST, as it failed to detect any of the cultured species in these samples. In contrast, Epi2me and Emu were able to detect at least one of the cultured species in a sample.

**FIGURE 4:**
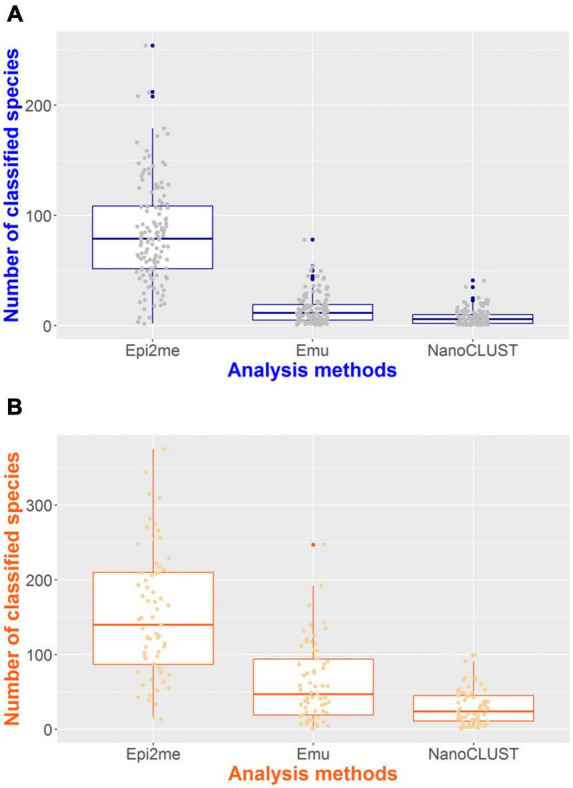
The number of classified species per sample by the three analysis pipelines in panel **(A)** monomicrobial samples and **(B)** polymicrobial samples.

The number of classified species per sample for each pipeline was summarized in Figure 4. In monomicrobial samples, despite single species being cultured, Nanopore 16S often classified more than one species (Figure 4A), regardless of the analysis pipelines. Additional organisms, apart from the cultured species, were detected in 128 (100%) samples, 122 (95.3%) samples, and 106 (82.8%) samples by Epi2me, Emu, and NanoCLUST, respectively. The average number of classified species per sample in monomicrobial samples was 83.9, 15.1, and 7.7 for Epi2me, Emu, and NanoCLUST, respectively. Interestingly, the cultured species were not necessarily the most abundant species in the correctly detected samples. In Epi2me, out of the 109 correctly detected samples, the cultured species in 27 samples were not the classified species with the highest relative abundance, including 3 samples with extremely low relative abundances (<0.1%). Similarly, in Emu, 37 out of 125 correctly detected samples were not the most abundant species among the classified species, and one sample had an extremely low relative abundance (<0.1%). For NanoCLUST, 25 out of 102 correctly detected samples were not the most abundantly classified species.

In the case of polymicrobial samples, the number of classified species per sample was significantly higher (*p* < 0.0001) compared to monomicrobial samples, regardless of the taxonomic classifiers used (Figure 4B). Similarly, Epi2me exhibited the highest number of classified species per sample, with an average of 153 species classified per sample, compared to 62 and 30 classified species in Emu and NanoCLUST, respectively. Additional organisms, beyond the expected ones, were detected in 65 (100%), 64 (98.5%), and 62 (95.4%) samples in Epi2me, Emu, and NanoCLUST, respectively. Notably, in Epi2me, 32 out of 50 (64.0%) concordant samples included species with extremely low relative abundances (<0.1%). In contrast, in Emu and NanoCLUST, 12 out of 37 (32.4%) and 1 out of 30 (3.33%) concordant samples included species with extremely low relative abundances, respectively. Therefore, Epi2me demonstrated a higher sensitivity for detecting extremely low abundance species.

### 3.4 Differentiation of *E. coli* and *Shigella* by the three pipelines

Among the three taxonomical classifiers, Emu showed the highest concordance with the reference culture method as it correctly differentiated *E. coli* from *Shigella* and other *Escherichia* species. On the other hand, both Epi2me and NanoCLUST misidentified *E. coli* as other closely related species in 12 monomicrobial samples. In polymicrobial samples, Epi2me misidentified *E. coli* in 37 samples, while NanoCLUST misidentified *E. coli* in 35 samples. It should be noted that in Epi2me, most of the reads of *E. coli* were classified at the family level (*Enterobacteriaceae*), resulting in a lower relative abundance of *E. coli* or related species at the species level compared to the results of Emu and NanoCLUST.

*E. coli* and *Shigella* are known to be closely related species with highly similar 16S rRNA gene sequences (Devanga Ragupathi et al., 2018). Although Emu outperformed the other two pipelines in identifying *E. coli*, their performance in identifying *Shigella* species was unknown due to the absence of *Shigella* species in the collected body fluids. To assess their ability to differentiate *E. coli* and *Shigella*, Nanopore 16S was performed using isolates of *E. coli*, *S. sonnei*, and *S. flexneri*, and the sequencing reads were analyzed using the three pipelines.

As shown in Table 1, both Epi2me and NanoCLUST could not differentiate *E. coli* and the two *Shigella* species, as they resulted in the same classified species, *E. fergusonii*. Furthermore, the relative abundance of *E. fergusonii* was much lower in Epi2me as most of the reads were classified only at the family level. In contrast, Emu correctly differentiated the three species.

**TABLE 1:** The analysis results of the three pipelines in differentiating *E. coli* and *Shigella* species.

Bacterial isolates	Total no. of reads	Epi2me		Emu		NanoCLUST	
		1st classified species	Relative abundance	1st classified species	Relative abundance	1st classified species	Relative abundance
*E. coli*	29,464	*E. fergusonii*	0.217	*E. coli*	0.999	*E. fergusonii*	1
*S. sonnei*	29,701	*E. fergusonii*	0.205	*S. sonnei*	0.749	*E. fergusonii*	1
*S. flexneri*	23,757	*E. fergusonii*	0.180	*S. flexneri*	0.986	*E. fergusonii*	1

### 3.5 Average number of classified species in different types of body fluids

Table 2 presents the average number of classified species obtained from Nanopore 16S coupled with Epi2me, Emu, and NanoCLUST, respectively, for various types of body fluids. Overall, ascitic fluid and peritoneal fluid exhibited a relatively higher average number of classified species compared to other body fluids, with most of these species belonging to the gut microbiota. It is worth noting that most of the polymicrobial body fluids observed in this study were ascitic fluid and peritoneal fluid. However, these gut microbes are often overlooked in culture-based methods since it is impractical to identify numerous gut microbes through culture.

**TABLE 2:** The average number of classified species by the three analysis pipelines in different types of body fluids.

	Specimen type	Epi2me	Emu	NanoCLUST
Monomicrobial	Abscess (*n* = 1)	21	3	2
	Ascitic fluid (*n* = 7)	65.6	15.1	7.7
	Cerebrospinal fluid (*n* = 2)	113	5.5	1.5
	Joint fluid/aspirate (*n* = 19)	85	13	5.7
	Pericardial fluid (*n* = 1)	23	18	9
	Peritoneal fluid (*n* = 16)	89.8	16.3	10
	Peritoneal dialysis fluid (*n* = 66)	81.1	15.2	7.9
	Pleural fluid/aspirate (*n* = 16)	100.7	17.4	8.2
Polymicrobial	Ascitic fluid (*n* = 10)	197.4	70.5	29.7
	Bile (*n* = 1)	190	25	14
	Intrauterine Fluid (*n* = 1)	123	9	5
	Midline laparotomy fluid (*n* = 1)	248	38	16
	Miscellaneous (*n* = 1)	178	36	19
	Peritoneal fluid (*n* = 38)	156.1	79.5	39.1
	Peritoneal dialysis fluid (*n* = 11)	112.5	14	6.8
	Pleural fluid (*n* = 2)	34	22.5	12.5
Culture-negative	Ascitic fluid (*n* = 3)	18.7	17.3	7.5
	Cerebrospinal fluid (*n* = 1)	8	7	N/A
	Joint fluid (*n* = 5)	32.6	17.8	5.7
	Lung abscess aspirate (*n* = 1)	81	8	4
	Pericardial fluid (*n* = 1)	N/A	N/A	N/A
	Peritoneal fluid (*n* = 1)	11	11	3
	Peritoneal dialysis fluid (*n* = 2)	56	21.5	11.5
	Pleural fluid/aspirate (*n* = 6)	42.8	13.3	7

### 3.6 Determination of threshold of relative abundance for detecting potential pathogens

**FIGURE 5:**
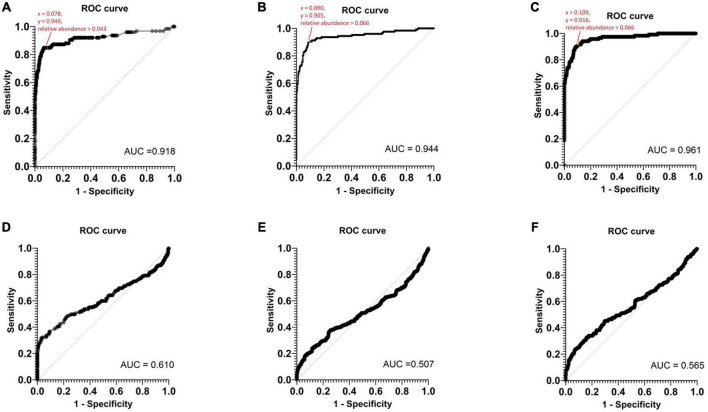
The ROC curves of monomicrobial samples based on panel **(A)** Epi2me, **(B)** Emu, **(C)** NanoCLUST analysis and the ROC curves of polymicrobial samples based on panel **(D)** Epi2me, **(E)** Emu, **(F)** NanoCLUST analysis.

A ROC analysis was conducted to establish the threshold of relative abundance for detecting potential pathogens using Nanopore 16S (Figure 5). In monomicrobial samples, the determined T_*RA*_ for Epi2me, Emu, and NanoCLUST were 0.043, 0.066, and 0.066, respectively, with a sensitivity and specificity equal to or greater than 90%. The average threshold across the three analysis pipelines was considered the Nanopore 16S threshold, which was 0.058. Applying this threshold correctly identified 107 (83.6%), 117 (91.4%), and 114 (91.2%) monomicrobial samples for Epi2me, Emu, and NanoCLUST, respectively. Notably, this included 20 (15.6%), 29 (22.7%), and 23 (18.0%) samples that were not identified as the most abundant species in Epi2me, Emu, and NanoCLUST, respectively.

However, T_*RA*_ could not be determined in the ROC analysis of polymicrobial samples. Nanopore 16S appeared to function as a random classifier in polymicrobial samples as numerous species exclusively detected by Nanopore 16S were considered “false positives” when compared to the culture results. Nevertheless, these “false positives” may actually be present in the body fluids but overlooked in culture-based methods. If the threshold of 0.058 was applied to the polymicrobial samples, the total number of cultured species detected decreased to 46 (20.0%), 59 (25.7%), and 64 (27.8%) for Epi2me, Emu, and NanoCLUST, respectively. This suggests in polymicrobial samples, most species had a relative abundance below the threshold of 0.058, which could be attributed to the presence of dominant species or the high complexity of the bacterial population in the sample.

### 3.7 Clinically important species found in culture-negative samples by Nanopore 16S

When applying the T_*RA*_ of 0.058, 13 out of the 20 culture-negative body fluids revealed the presence of clinically important species, including some opportunistic pathogens. The sequencing results were summarized in Supplementary Table 6. The clinical background of the patients was examined for these thirteen Nanopore 16S-positive samples (Table 3). Particularly, the inferred species from the Nanopore 16S results corresponded to the patients’ medical history in two samples (19MB090618 and 20MB068970). In the case of 19MB090618, *Streptococcus pneumoniae* was inferred by Nanopore 16S, which matched the detection of pneumococcal antigen in urine samples. Similarly, *Prevotella* detected by Nanopore 16S in 20MB068970 correlated with the isolation of *Prevotella* from a previous sample. The main reason for the inability to detect the Nanopore 16S inferred species in culture was the prescription of empirical antibiotics. Out of the thirteen patients, eleven had been prescribed antibiotics prior to sample collection. However, for the two samples without antibiotic treatment (21M2019392 and 21M2019576), the positive Nanopore 16S results were likely due to contamination from skin flora. In the case of 21M2019576, the detection of E. coli was also expected considering the presence of a duodenal ulcer.

**TABLE 3:** The clinical background of thirteen culture-negative samples with positive Nanopore 16S result.

Sample ID	Specimen type	Bacteria detected by Nanopore 16S	Clinical detail/Diagnosis	Previous culture result	Pre-treatment before sample collection
19B2153436	Pleural aspirate	*Streptococcus intermedius*	Right pleural effusion and empyema, cough, SOB and chest discomfort for 2 weeks.	No history of the isolate	Yes. Tazocin and Ornidazole.
		*Parvimonas micra*			
19B2153759	Pleural aspirate	*Parvimonas micra*	Right pleural effusion, under chemotherapy, right breast cancer with high suspicion of multiple vertebral metastases.	No history of the isolate	Yes. Tazocin and Vancomycin.
		*Streptococcus milleri/* *Streptococcus constellatus*			
		*Escherichia coli*			
20MP2015461	Knee joint fluid (left)	*Enterococcus cecorum*	Gout	No history of the isolate	Yes. IV Augmentin.
		*Staphylococcus aureus*			
20MP2015462	Pleural fluid	*Facklamia hominis*	TB-pericarditis	No history of the isolate, but AFB positive in pericardial fluid	Yes. PO/IV Augmentin.
21M2019392	Ascitic fluid	*Staphylococcus hominis*	Malignancy-related ascites	No history of the isolate	No.
21M2019256	Peritoneal dialysis fluid	*Moraxella osloensis*	Fluid overload	No history of the isolate	Yes. IV Augmentin.
21M2019465	Peritoneal fluid	*Cutibacterium acnes*	Fever, malignancy-related ascites	No history of the isolate	Yes. IV Augmentin.
		*Staphylococcus hominis*			
21M2019576	Knee joint fluid (left)	*Moraxella osloensis*	Fever, duodenal ulcer	No history of the isolate	No.
		*Escherichia coli*			
19MB068751	Joint fluid (right shoulder)	*Staphylococcus epidermidis*	Septic arthritis	No history of the isolate	Yes. IV Augmentin.
		*Staphylococcus hominis*			
		*Staphylococcus cohnii*			
18MB084305	Joint fluid	*Streptococcus canis*	Prosthetic joint infection	No history of the isolate	Yes. IV Tazocin.
19MB090618	Lung abscess aspirate	*Streptococcus pneumoniae*	Community acquired pneumonia with lung abscess	No history of isolate, but urine pneumococcal antigen positive.	Yes. IV Augmentin.
19MB069360	Ascitic fluid	*Escherichia coli*	Peritonitis	No history of the isolate	Yes. IV Augmentin.
20MB068970	Pleural fluid	*Prevotella oris*	Perforated esophagus with lung empyema	Previous pleural fluid culture: *Enterococcus faecalis, Lactobacillus* species, *Candida albicans, Prevotella* species	Yes. IV vancomycin, IV meropenem
		*Streptococcus anginosus*			

### 3.8 LOD of Nanopore 16S

**FIGURE 6:**
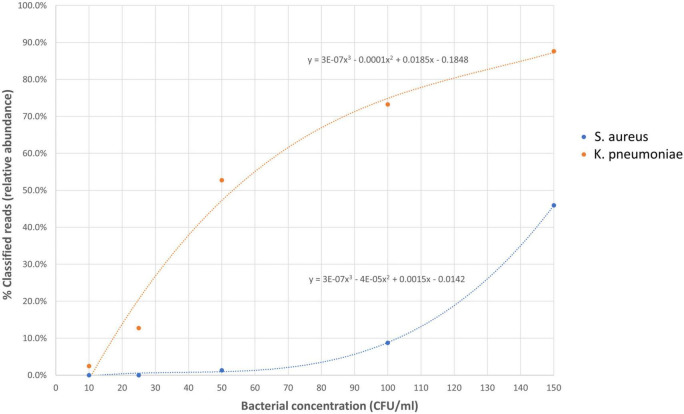
The relative abundance of spiked species in Nanopore 16S against the bacterial concentration in simulated bacteremic blood.

To determine the limit of detection (LOD) of Nanopore 16S, one Gram-positive (*S. aureus*) and one Gram-negative (*K. pneumoniae*) pathogen, commonly found in body fluids, were spiked in EDTA-blood at various dilutions (Figure 6). During the LOD test, Nanopore 16S showed sensitivity to environmental contamination when the bacterial load in the sample was extremely low. Consequently, some environmental bacteria such as *Pelomonas saccharophila*, *Cutibacterium acnes*, and *Stenotrophomonas rhizophila* were detected. Based on the T_*RA*_ calculated from the ROC analysis (0.058), the LOD of Nanopore 16S for *S. aureus* and *K. pneumoniae* was determined to be 89.32 CFU/ml and 14.47 CFU/ml, respectively. Therefore, the overall LOD was approximately 90 CFU/ml.

### 3.9 Workflow of Nanopore 16S

**FIGURE 7:**
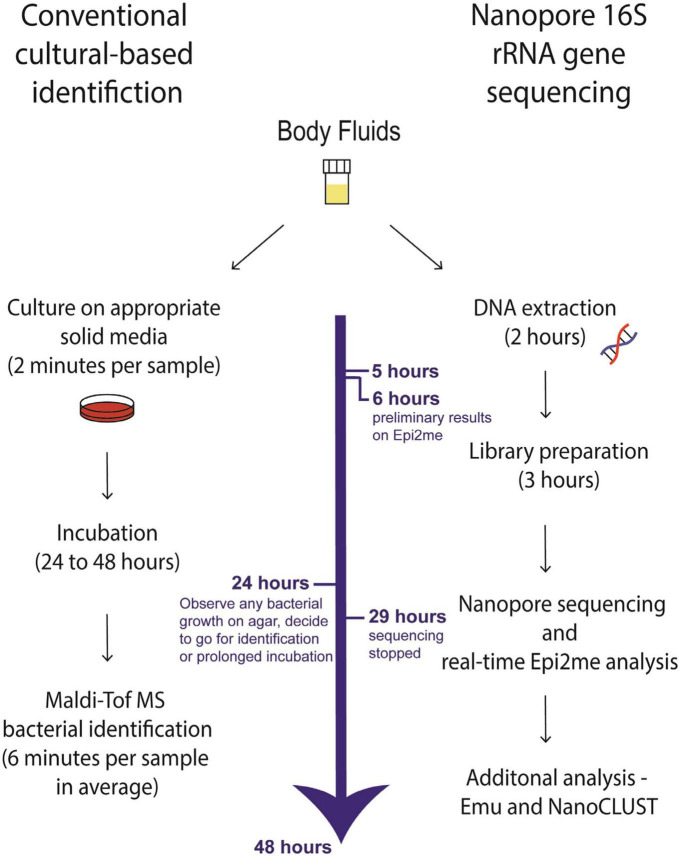
The workflow of cultural-based identification and Nanopore 16S.

Figure 7 illustrates the workflow of Nanopore 16S compared to conventional culture-based identification. The culture-based identification typically requires at least 24 h to obtain results, with even longer timeframes for fastidious bacteria. In contrast, Nanopore 16S coupled with Epi2me provides preliminary results within just 6 h. Among the 127 concordant monomicrobial samples (including closely related species) analyzed using Nanopore 16S coupled with Epi2me, all targeted species or closely related species were detected within the first hour of sequencing (Supplementary Table 7). Among the 50 concordant polymicrobial samples (including closely related species), 31 samples (62%) showed detection of all targeted species within 1 h of sequencing using Nanopore 16S coupled with Epi2me. Since the bacterial load varies greatly among the samples, the sequencing time was set to 24 h in this study to increase the sequencing depth of samples with extremely low DNA concentration. However, the sequencing time can be significantly shortened since the targeted species could be detected within the first few hours in most of the samples.

## 4 Discussion

Although culture-based identification is commonly used for routine bacterial identification in clinical laboratories, there are still some limitations. Apart from the lengthy sample-to-report time, culture-based methods provide an incomplete bacterial profile of the samples. Apart from the uncultivable bacteria, anaerobic bacteria are often overlooked in culture-based approaches (Nagy et al., 2018; Cummings et al., 2020). Moreover, it is not uncommon that patients may have received empirical antibiotic treatment prior to their hospital admission. As a result, the causative agent may have been eradicated or suppressed, leading to negative culture results. In such cases, the Nanopore 16S analysis can still identify and detect dead microorganisms, which can provide valuable insights into the potential pathogens involved. This capability helps to uncover the underlying pathogen and can aid in resolving undiagnosed cases that may have been missed by culture-based methods.

In this study, the performance of Nanopore 16S for bacterial identification in direct body fluids was evaluated, with culture standards as a reference. In addition to the Epi2me from ONT, the sequencing reads were also analyzed using two external analysis pipelines, Emu and NanoCLUST, to reduce the classification bias. Nanopore 16S showed the highest concordance when coupled with Emu, with concordance rates of 97.7 and 81.7% for monomicrobial and polymicrobial samples, respectively. However, Epi2me showed the highest concordance when closely related species were included, with concordance rates of 99.2 and 90.0% for monomicrobial and polymicrobial samples, respectively. NanoCLUST had the lowest concordance among the three analysis pipelines, with rates of 93.0% for monomicrobial samples and 79.89% for polymicrobial samples, including closely related species.

The high sensitivity of Epi2me was attributed to its read-by-read classification approach. However, this approach is prone to sequencing errors in individual reads, which can result in misassignment of taxa to closely related species and an increased number of classified species per sample. It is possible to reduce the number of classified species per sample in Epi2me by increasing the percentage similarity, however, the sensitivity will decrease as well. In contrast, NanoCLUST had the lowest sensitivity among the three analysis pipelines, as its clustering approach for highly similar sequences led to a loss of sensitivity in detecting closely related and low-abundance species. Emu, which involves read alignment with reference sequences followed by an error-correction step based on an expectation–maximization algorithm, highly effective at distinguishing closely related species (Curry et al., 2022), demonstrated a better balance in terms of sensitivity and the number of classified species.

Nanopore 16S typically identified a higher number of species compared to culture, especially in polymicrobial samples, regardless of the analysis pipeline used. Additionally, the cultured species in monomicrobial samples were not necessarily the most abundant species detected in Nanopore 16S. This disparity can be attributed to the presence of contaminants in Nanopore 16S results or the under-detection of microorganisms in culture. Body fluids with a low bacterial load were particularly susceptible to environmental DNA contamination, especially when sterilization processes like autoclaving were insufficient to remove DNA (Suyama and Kawaharasaki, 2013; Yap et al., 2013) from surgical and laboratory equipment. Environmental bacteria, such as *Aquitalea magnusonii*, *Delftia acidovorans*, and *Deinococcus geothermalis*, were found in certain body fluids in this study. Besides, anaerobes were often missed in culture, and some gut microbiota, including *Parvimonas micra*, *Peptostreptococcus stomatis*, *Faecalibacterium prausnitzii*, *Fusobacterium nucleatum*, and *Filifactor alocis*, were detected by Nanopore 16S but seldom reported by culture. Conversely, there were some species that were only detected by culture and not by Nanopore 16S, possibly because the targeted species were masked by other abundant species in the samples.

Since Nanopore 16S is sensitive to contamination, some measures were taken to avoid sample contamination during DNA extraction and library preparation. During the transportation, each sample was kept in separated ziplock bag to avoid cross-contamination. DNA extraction was performed in a class II biosafety cabinet to prevent environmental contamination. All the equipment was cleaned with 10% bleach and 70% ethanol before and after the extraction. In addition, the gloves were wiped with 10% bleach each time before handling the next sample to reduce the chance of cross-contamination between samples during the extraction. Library preparation which involved handling of PCR amplicons was conducted in a post-PCR area to prevent contamination of the next batch of samples with 16s rDNA amplicons. In each sequencing batch, nuclease-free water was used as non-template control (NTC) to detect the environmental and kit contaminants. Any reads identified in the NTC could be considered as contaminants and excluded from the samples if detected.

Receiver operating characteristic analysis was performed to determine the threshold of relative abundance for differentiating potential pathogens from the background. The ROC analysis from monomicrobial samples suggested that a threshold of 0.058 could achieve at least 90% sensitivity and specificity for Nanopore 16S. Applying this threshold, the LOD of Nanopore 16S was determined to be 89.32 CFU/ml for *S. aureus* and 14.47 CFU/ml for *K. pneumoniae*. The higher LOD for Gram-positive species compared to Gram-negative species may be attributed to the thicker cell wall of Gram-positive bacteria, which makes them more difficult to lyse during extraction steps (Li et al., 2020). Therefore, the overall LOD of Nanopore 16S was approximately 90 CFU/ml.

The main limitation of 16S rRNA gene sequencing is that it focuses on bacterial identification, but provides no information about the functional genes in the microbial community and is unable to identify other pathogens like fungi and viruses (Durazzi et al., 2021). Although sequencing the entire 16S rRNA gene could improve the taxonomic resolution at the species level, some closely related species with highly similar 16S rRNA gene sequences might not be well differentiated. Comparatively, metagenomic sequencing, which captures entire genetic material in the sample, enables detection of all kinds of pathogens and the functional genes. It also provides better taxonomic resolution since the entire genome of the microbe was sequenced. However, metagenomic sequencing is less sensitive than the 16S rRNA gene sequencing since the presence of host DNA significantly lowers the sequencing depth of microbial DNA (Beaudry et al., 2021). Moreover, the metagenomic workflow will be more tedious than the Nanopore 16S workflow. Additional host DNA depletion is required during DNA extraction and fragmentation of genomic DNA is required in library preparation.

There were limitations in this study. Culture was used as the only reference standard for evaluating the performance of Nanopore 16S, as there is no perfect reference method that truly reflects the bacterial profile of body fluids. Numerous species exclusively detected by Nanopore 16S were considered “false positives” when compared to the culture results. Nevertheless, these “false positives” may actually be present in the body fluids but overlooked in culture-based methods. Consequently, the threshold of relative abundance could not be determined from the ROC analysis of polymicrobial samples. Furthermore, the sample size for each type of body fluids was uneven, with half of them being peritoneal fluids or peritoneal dialysis fluids.

Nanopore 16S allows rapid diagnosis, particularly in medical emergencies, but it should not be solely relied upon for clinical diagnosis. While providing a more complete bacterial profile, Nanopore 16S only suggests potential causative agents. The choice of classifiers can also lead to differences in bacterial profile. Accurate diagnosis relies on integrating laboratory findings with the patient’s medical history, clinical manifestations, and expertise of clinicians. For instance, the presence of normal flora in body fluids may indicate incidental contamination during sample collection or an opportunistic pathogen. Concurrent culture-based identification can help to detect low-abundance organisms below the LOD of Nanopore 16S.

## Data availability statement

The datasets presented in this study can be found in online repositories. The names of the repository/repositories and accession number(s) can be found below: https://www.ncbi.nlm.nih.gov/, PRJNA1009267.

## Ethics statement

The studies involving humans were approved by the New Territories West Cluster Research Ethics Committee, the Kowloon West Cluster Research Ethics Committee, the Joint Chinese University of Hong Kong-New Territories East Cluster Clinical Research Ethics Committee, and the Hong Kong East Cluster Research Ethics Committee. The studies were conducted in accordance with the local legislation and institutional requirements. The human samples used in this study were acquired from the clinical laboratories of four local hospitals: Tuen Mun Hospital, Princess Margaret Hospital, Prince of Wales Hospital, and Pamela Youde Nethersole Eastern Hospital. Written informed consent for participation was not required from the participants or the participants’ legal guardians/next of kin in accordance with the national legislation and institutional requirements.

## Author contributions

H-YL: Formal analysis, Investigation, Methodology, Visualization, Writing—original draft. LW: Investigation, Writing—review and editing. YH: Investigation, Writing—review and editing. TN: Formal analysis, Methodology, Software, Writing—review and editing. CC: Formal analysis, Writing—review and editing. HL: Investigation, Writing—review and editing. MY: Data curation, Resources, Writing—review and editing. EL: Data curation, Resources, Writing—review and editing. RW: Data curation, Resources, Writing—review and editing. AH: Data curation, Resources, Writing—review and editing. K-TY: Data curation, Resources, Writing—review and editing. JL: Data curation, Resources, Writing—review and editing. VC: Data curation, Resources, Writing—review and editing. KL: Data curation, Resources, Writing—review and editing. T-LQ: Data curation, Resources, Writing—review and editing. FC: Writing—review and editing. GS: Conceptualization, Funding acquisition, Methodology, Project administration, Supervision, Writing—review and editing.
